# Editorial: New avenues of silicon’s role in plant biology: trends and controversies

**DOI:** 10.3389/fpls.2025.1717302

**Published:** 2025-10-29

**Authors:** Mohammad Mukarram, Francisco J. Corpas, Alexander Lux

**Affiliations:** ^1^ College of Plant Protection, Jilin Agricultural University, Changchun, China; ^2^ Antioxidant, Free Radical and Nitric Oxide in Biotechnology, Food and Agriculture Group, Estación Experimental del Zaidín, Consejo Superior de Investigaciones Científicas (CSIC), Granada, Spain; ^3^ Department of Plant Physiology, Faculty of Natural Sciences, Comenius University in Bratislava, Bratislava, Slovakia; ^4^ Institute of Chemistry, Slovak Academy of Sciences, Bratislava, Slovakia

**Keywords:** silicon, abiotic stress, oxidative damage, silicon nanoparticle, SiNPs, beneficial elements

## 1 Silicon paradox in plant biology

Silicon (Si) is a paradoxical element in plant biology: abundant in soils yet historically omitted from essential nutrient lists (Epstein, 1999). Over the past few decades, evidence has established Si as a *quasi-essential* element that can enhance plant growth, yield, and resilience under stress (Manivannan et al., 2023). Several Si-accumulating plants, such as *Oryza sativa*, uptake astonishing amounts of Si (up to ~10% of their dry weight, exceeding even macronutrients like N or K) (Hodson et al., 2005). Yet how Si confers these advantages remains an active area of debate. Is Si primarily fortifying cell walls and acting as a mechanical barrier, or is it integral to plant stress physiology? This core controversy of anatomical *vs* physiological modes of Si action is a rapidly evolving frontier. Researchers are probing Si’s *modus operandi*, crosstalk with signalling molecules (e.g., reactive oxygen species (ROS) and phytohormones), and its stress-mitigating potential. The present Research Topic aims to advance our understanding of these phenomena. Articles in this Research Topic tackle Si from diverse angles, ranging from stress mitigation and nutrient dynamics to molecular mechanisms and innovative Si delivery methods, reflecting the breadth of current Si research.

## 2 Silicon in environmental stress mitigation

In this Research Topic, Ansari et al. probed how Si application mitigated Cd- and Pb-induced oxidative burst (MDA, H_2_O_2_, electrolyte leakage) and metal uptake in *Capsicum annuum*. Possible mechanistic reasons may include metal immobilisation, altering uptake kinetics, or even influencing signal proteins to regulate Si uptake (de Tombeur et al., 2021; Mukarram et al., 2024; Yamaji et al., 2024). Also, these plants had enhanced antioxidant enzymes. Further, Tritean et al. embedded rice husk silica nanoparticles (SiNPs) in an alginate seed film to create a slow-release Si source. SiNPs-coated *Vigna radiata* seeds exhibited more vigorous early growth than controls under high salt conditions, owing to enhanced metabolic activity, proton pump function, and ROS scavenging in the seedlings. This seed priming strategy with Si opens new avenues for protecting crops against stress right from the outset.

## 3 Silicon and nutrient dynamics

He et al. present field evidence that moderate (750–1500 kg/ha) Si fertilisation can enhance both the yield and quality of a high-value crop (*Nicotiana tabacum*). Si increased the N and K use efficiency by up to p~40%, having clear agronomic significance for reducing fertiliser waste and environmental runoff. Si-treated leaves had higher sugar-to-alkaloid ratios and mineral balance (higher desirable K, lower Cl). These modulations resulted in the increased high-grade *Nicotiana tabacum* leaves (~95%), yield (15.7%), and economic output (30.8%) over no Si application. However, the highest dose (3000 kg/ha) slightly depressed leaf quality by elevating nicotine and upsetting the sugar-alkali balance. This dose-dependent response underscores the need to optimise Si nutrition, as more is not always better. He et al.’s findings align with a broader recognition that Si can improve plant nutrient status, better resource use, and product quality (Pavlovic et al., 2021; Schaller et al., 2024).

## 4 Silicon’s *modus operandi*

**Figure 1:**
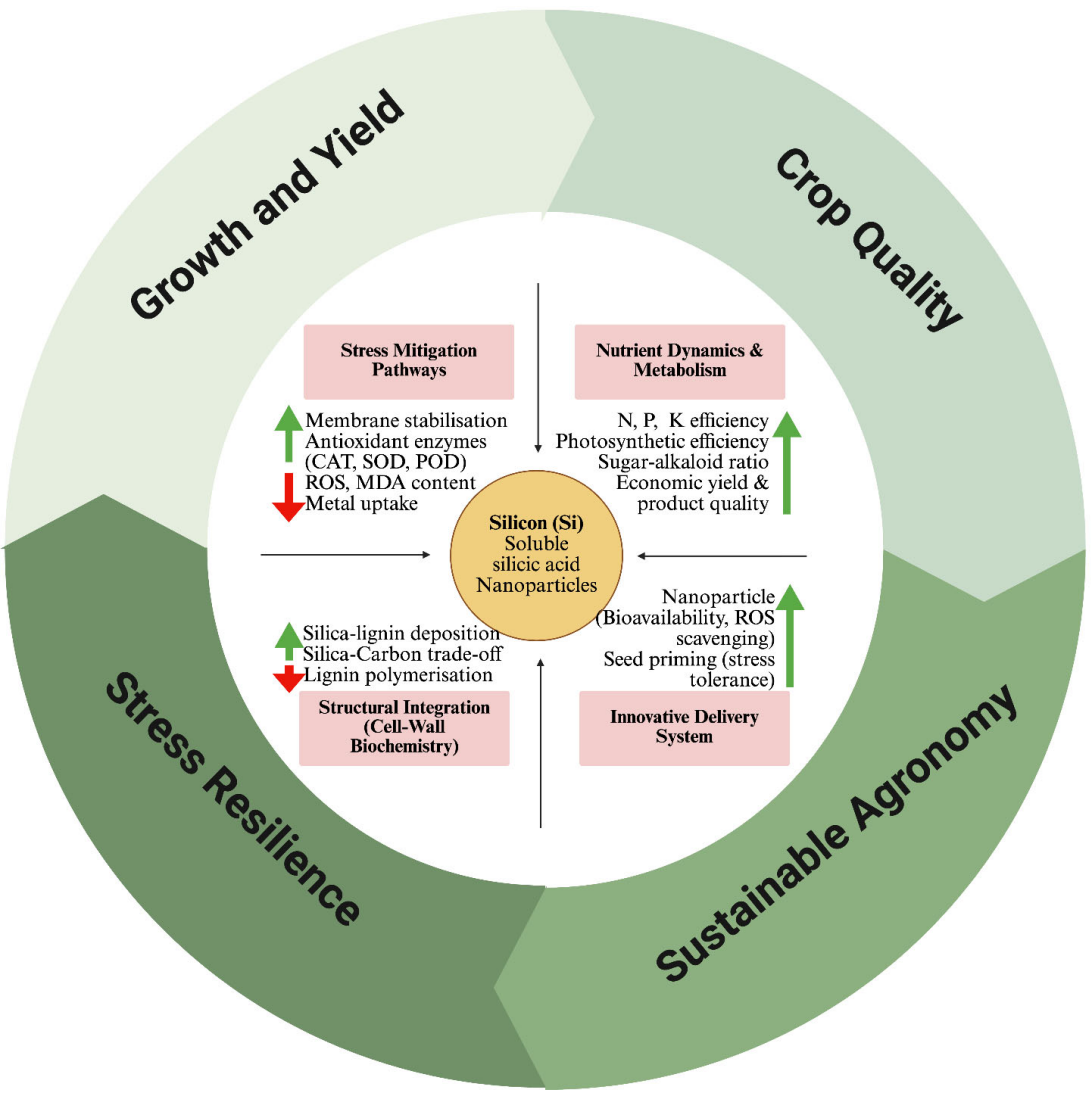
Biochemical and physiological pathways of silicon (Si) in plants. Si enhances plant resilience through stress mitigation, improved nutrient use, and structural reinforcement of cell walls, while innovative delivery systems improve Si efficacy. These combined effects promote growth, crop quality, and sustainable agriculture. Green upward arrows indicate upregulation/increment, while the red downward arrows indicate downregulation/reduction in the adjacent phenomena/process. CAT, catalase. POD, peroxidase; SOD, superoxide dismutase; MDA, malondialdehyde; ROS, reactive oxygen species (Created in https://BioRender.com).

What mechanistic magic underlies silicon’s broad benefits? Zexer et al. revealed that silica aggregates alongside the short-chain, less-polymerised lignin monomers in root endodermal cell walls. Alternatively, the lignin at these sites polymerises into more extensive, highly cross-linked doughnut-shaped structures in Si-absence. The authors suggested that silicic acid interrupts the typical polymerisation of lignin and nucleates silica gel. This finding reinforces the hypothesised “Si-C trade-off” in plant defence strategies (de Tombeur et al., 2025). Si seems to reallocate costly carbon-based compounds (like lignin, phenolics, or tannins) with Si deposits as a more energy-efficient means of fortifying tissues. On the physiological side, Ansari et al. noted Si’s passive exclusion of toxins and its active induction of defence genes. Increased activities of SOD, CAT, and other enzymes in Si-treated plants are a common observation under various stresses (Ansari et al.; Yan et al., 2023). Si has further been reported to regulate signalling molecules (H_2_O_2_ and NO) to prime plant stress response (Mukarram et al., 2022) (**Figure 1**).

## 5 Advances with silicon nanoparticles (SiNPs)

Recent advancements include the development of SiNPs that offer greater effectiveness compared to traditional silicate fertilisers (Mukarram et al., 2022; Yan et al., 2023). SiNPs have a high surface area and reactivity that can improve the Si bioavailability to plants. Studies summarised by Yan et al. show that exogenous SiNPs can penetrate plant tissues more readily than bulk Si sources. SiNPs have been shown to reduce ROS levels and upregulate antioxidant enzymes (CAT, SOD, and POD) in stressed plants more than equivalent doses of conventional silicate salts (Mukarram et al., 2023; Yan et al.). These remarkable effects underscore that *size matters*.

## 6 Some key unanswered questions

While Si effects are well reported, we still seek to understand several key phenomena. For example, (1) how are Si effects realised at the molecular level (e.g., signalling networks for H_2_O_2_, NO, or phytohormones)? (2) Do plants actively sense Si, or are its effects primarily indirect via stress alleviation? (3) What is Si’s primary mode of action (structural *vs* metabolic)? (4) What are the long-term and ecosystem-level impacts of heavy/continued Si use? How does Si supplementation affect the soil cycle? Could there be unintended ecological effects or diminishing returns over time? Furthermore, (5) standardised protocols (e.g., for Si depletion in growth media, or for quantifying plant-available Si) should be developed to reduce variability across studies and ensure reproducibility. (6) Si research should be expanded to more plant types (non-accumulators, wild species, ecological communities) to uncover new Si facets and validate their generality. (7) Precision Si delivery systems and breeding crops with enhanced Si uptake are interesting frontiers to develop.

As the plant science community addresses the open questions and refines methodologies, we anticipate rapid progress in demystifying how Si works and how best to harness it. We hope this Research Topic will inspire cross-disciplinary collaboration and many other interesting Si studies.
